# Experiences of Anxiety and Its Relationship to Freezing of Gait in Parkinson's Disease: A Qualitative Study

**DOI:** 10.1111/ejn.70581

**Published:** 2026-06-17

**Authors:** Gijs Vissers, William R. Young, Jorik Nonnekes

**Affiliations:** ^1^ Department of Rehabilitation, Donders Institute for Brain, Cognition and Behavior Radboud University Medical Center Nijmegen the Netherlands; ^2^ School of Sport and Health Sciences University of Exeter Exeter UK

**Keywords:** anxiety, freezing of gait, Parkinson's disease, qualitative research, thematic analysis

## Abstract

Freezing of gait (FOG) is a disabling motor symptom in Parkinson's disease (PD) with substantial impact on mobility and quality of life. Anxiety is frequently implicated in triggering or worsening FOG, yet the lived experience of this interaction remains poorly understood. This study explored how individuals with PD reflect on the interaction between anxiety and FOG following an anxiety‐focused intervention, aiming to better understand how and why this interaction develops and impacts daily life. A thematic analysis was conducted using semistructured interviews with 12 individuals with PD who reported daily FOG episodes influenced by anxiety or stress. Interviews were conducted during the final session of a behavioral intervention targeting anxiety related to FOG. Interviews were transcribed verbatim and analyzed iteratively to identify patterns of meaning. Five overarching themes emerged. First, participants described how anxiety was linked to a gradual loss of confidence in their walking abilities, driven by perceived loss of control, unpredictability of FOG, and feelings of vulnerability. Second, regaining control over walking was mentioned to reduce anxiety, either through having a compensatory strategy available in case of a freezing episode, or through contextual factors (e.g., being in the dopaminergic ON state). Third, anxiety typically developed gradually alongside the progression of FOG and the concomitant decline in confidence during walking, but could also intensify following negative events, such as falls. Fourth, anxiety was reported to divert attentional resources toward potential threats and make it more difficult to focus on strategies for managing FOG. Finally, many participants engaged in avoidance behaviors, causing negative downstream effects such as limiting social participation and physical activity. These findings highlight the importance of early recognition, as well as the potential for interventions that enhance confidence to help reduce anxiety surrounding FOG.

## Graphical abstract

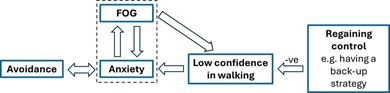

**Abbreviations**
%FOG: mean percentage of time frozen during a personalized home‐based walking trajectory that was part of the TACKLING‐FOG trial
FOG: Freezing of Gait
MDS‐UPDRS: Movement Disorders Society—Unified Parkinson's Disease Rating Scale
MoCA: Montreal Cognitive Assessment
NFOG‐Q: New Freezing of Gait Questionnaire
NRS: numeric rating scale (0–10) indicating to what extent anxiety or stress affected FOG in the past week
PD: Parkinson's Disease

## 1 Introduction

Freezing of gait (FOG) is one of the most debilitating motor symptoms in Parkinson's disease (PD), characterized by paroxysmal episodes where there is an inability to step effectively, despite attempting to do so (Weiss et al. 2020). FOG has a profound impact on the quality of life of people with PD, as it severely limits their daily mobility and is a major contributor to falls and fall‐related injuries (Walton et al. 2015; Canning et al. 2014). It is relatively common, with up to 80% of people with PD experiencing FOG in the later stages of the disease (Macht et al. 2007). FOG frequently occurs during gait initiation, turning, or when performing a cognitive dual‐task (e.g., having a conversation while walking) (Conde et al. 2023).

In addition to these triggers, anxiety is a common psychological factor that is implicated in the occurrence and exacerbation of FOG (Martens et al. 2014; Martens et al. 2018; Taylor et al. 2022). Its influence is often apparent in situations where individuals anticipate a freezing episode, are concerned about falling, or experience time pressure (Cockx et al. 2023; Giladi and Hausdorff 2006; Uhlig and Prell 2023). Conversely, freezing episodes can also cause anxiety in people with PD, in part because FOG is a known risk factor for falls (Ghielen et al. 2020). Because of this bidirectional relationship, a vicious cycle may emerge where anxiety precipitates FOG, which in turn further increases anxiety. A possible mechanism by which anxiety might influence FOG is through its impact on attentional resources during walking. As people with PD increasingly have to rely on conscious control of gait to compensate for the loss of automatic gait regulation, anxiety might exacerbate FOG by redirecting attentional resources from conscious monitoring of movement (goal‐directed gait control) toward processing threat‐related stimuli (e.g., anxious thoughts about FOG or focusing on the area where one expects to freeze) (Eysenck et al. 2007; Rosenblum et al. 2025).

While quantitative studies have demonstrated associations between anxiety and FOG, they lack the ability to capture the subjective experiences and contextual factors that shape the interplay between anxiety and FOG. For example, how anxiety and FOG interact over the course of FOG progression remains unclear. Also, it is unknown whether anxiety surrounding FOG is continuously present or fluctuates from moment to moment, and if so, what factors contribute to these fluctuations. A qualitative approach can therefore provide a valuable addition to the literature by capturing rich, detailed accounts of how FOG‐related anxiety is experienced by people with PD. This can provide valuable insight into the mechanisms involved in the onset and development of FOG‐related anxiety, how people respond to perceived challenging situations that are related to FOG, and how it influences attentional focus during walking. This approach may inform recommendations to mitigate FOG‐related anxiety, thereby complementing the existing quantitative literature and contributing to a more comprehensive understanding of the interplay between anxiety and FOG.

Accordingly, this study aimed to address the following research question: How do individuals with PD reflect on the interaction between anxiety and FOG following an anxiety‐focused intervention?

## 2 Methods

Ethical approval was obtained from the Medical Ethics Committee Oost‐Nederland, the Netherlands, as part of the TACKLING‐FOG trial (Clinicaltrials.gov identifier: NCT06302309) and the research was carried out in accordance with the Declaration of Helsinki (Vissers et al. 2026).

### 2.1 Design

We conducted a qualitative study using thematic analysis to analyze participants' accounts of FOG‐related anxiety. The study was reported following the Standards for Reporting Qualitative Research (SRQR) (O'Brien et al. 2014).

### 2.2 Researcher Characteristics and Reflexivity

The primary researcher who conducted the interviews (GV) is a male PhD student conducting a clinical trial on the effectiveness of a behavioral intervention targeting anxiety‐ and stress‐related FOG, which has provided him with in‐depth knowledge of the topic. Co‐authors JN and WY, who are also involved in the clinical trial, are male senior researchers: JN is a consultant in rehabilitation medicine and associate professor with expertise in gait disorders in PD. WY is a psychologist and associate professor who focuses on the influence of anxiety and cognitive processes on balance and walking in both healthy populations and people with PD and has ample experience with qualitative research methods.

### 2.3 Participants

People with PD who experienced daily FOG related to anxiety or stress were purposively recruited as part of the larger TACKLING‐FOG trial (Vissers et al. 2026). Participants were recruited via the Parkinson recruiting platform ParkinsonNEXT, the research page of the Dutch Parkinson's patient organization, the outpatient clinic of the Centre of Expertise for Parkinson's disease, and from an existing cohort of previous study participants. Interested individuals received an information letter and were contacted by telephone after one week to confirm participation, after which eligibility was assessed. Participants were eligible if they had a diagnosis of idiopathic PD according to the MDS criteria (Postuma et al. 2015) and experienced FOG episodes multiple times per day (as assessed using the New Freezing of Gait Questionnaire) that were related to anxiety. Exclusion criteria included comorbidities (e.g., neurological or orthopedic conditions) that significantly affected gait or severe cognitive impairment.

Within this broader trial sample, a purposive subsample of 12 participants was selected for qualitative interviews. The interviews were introduced during a later phase of the study, once approximately half of the intervention participants had completed the program. Sampling aimed to include information‐rich cases in terms of participants' ability to reflect on and articulate their experiences with anxiety‐related FOG while ensuring variation in relevant clinical and demographic characteristics. This involved prioritizing participants who were considered able to reflect on and describe their experiences in sufficient detail during conversation, as this facilitated the collection of rich qualitative data. Importantly, selection was not based on participants' response to the intervention, as the aim of the qualitative study was to gain a deeper understanding of participants' experiences regarding the interplay between anxiety and FOG rather than to evaluate responses to the intervention. Data collection was stopped when no substantially new information was obtained from additional interviews.

### 2.4 Interviews

Individual, semistructured interviews were conducted either face‐to‐face in participants' homes or via a video‐call platform. Interviews were conducted with the participant alone or with their partner present. The interviews were conducted in Dutch, the native language of all participants. Questions were primarily directed towards participants themselves, but partners were also invited to contribute when relevant in order to provide additional contextual information. In cases where partners reported a different perspective from the participant, both perspectives were discussed. An interview guide was used to ensure that relevant topics were covered and to provide continuity throughout the conversation (Supporting Information). During the interview, follow‐up questions were asked based on the participants' responses. Interviews lasted between 28 and 44 min, with an average duration of 34 min. All interviews were audio‐recorded and transcribed verbatim. Initial reflections on relevant information and potential follow‐up questions were documented immediately after the interviews. Demographic characteristics of participants were collected as part of the TACKLING‐FOG trial (Vissers et al. 2026). Member checking was not performed.

As participants were also enrolled in the TACKLING‐FOG trial, they had prior contact with the interviewer (GV). This contact consisted of four sessions that lasted 60 to 90 min each. During these sessions, the researcher explained the relationship between anxiety and FOG, explored situations in which anxiety affected FOG, and introduced strategies to reduce anxiety, such as breathing exercises and positive affirmations, which were practiced and evaluated in the last three sessions. The interviews were conducted during the final session of the intervention, during a period in which participants had engaged with topics related to anxiety and FOG, allowing them to reflect on this relationship in relation to their personal experiences. This enabled participants to share and articulate their experiences regarding the relationship between anxiety and FOG in more depth. The timing of the interviews may have influenced participants' responses by shaping how they interpreted and described their experiences, which was addressed by also asking participants to reflect on their experiences prior to the intervention.

### 2.5 Analysis

Data were analyzed using ATLAS.ti version 24.0.0, ATLAS.ti Scientific Software Development GmbH, Berlin, Germany. A thematic analysis was conducted following the approach of Braun and Clarke (Braun and Clarke 2019), which provides a flexible yet structured framework for identifying patterns and themes of meaning. Relevant data were coded by the first author (GV) based on the topics defined in the interview guide. An interpretative coding approach was used to capture the underlying meanings and perspectives that were embedded in the data. The analysis followed an inductive approach, while remaining sensitized to specific topics of interest, namely attentional focus during anxiety related to FOG and the course of anxiety related to FOG. Following initial coding, related codes were grouped into preliminary themes. An iterative process of reviewing and refining the codes and themes was employed to ensure that the final themes accurately reflected the data. A concluding meeting with all authors was held to ensure consensus was reached on the final themes.

To gain a deeper understanding of the data, a research assistant (NL) independently coded four interviews, while the full dataset was solely coded by GV. GV and NL then held a meeting to discuss and reflect on their coding, thereby enhancing the credibility of the findings. In addition, to further enhance rigor and credibility, a peer debriefing session was conducted after the initial coding phase with WY, who was not otherwise involved in the actual data analysis, during which interpretations of the data were critically examined. Specifically, codes about which GV had doubts or questions were discussed jointly by reviewing the relevant sections of the transcripts on which the codes were based. Illustrative quotes included in the manuscript were translated from Dutch by GV. When there was uncertainty about the most appropriate wording or nuance, translations were discussed with WY, a native English speaker.

## 3 Results

### 3.1 Demographics and Clinical Characteristics

Participants were aged between 45 and 78 years (mean: 68.7 years), including 6 men and 6 women (see Table 1). All participants reported an increase in FOG due to anxiety at the start of participating in the intervention study (Numeric Rating Scale item [scale 0–10]: “To what extent did anxiety or stress impact the occurrence of FOG during the past week?” mean = 6.2, range = 3–9). Self‐reported FOG severity was high, as reflected by NFOG‐Q scores, with a mean of 20 and a range of 10 to 23. Disease severity, as measured by the UPDRS Part‐III, ranged from 31 to 53, with a mean score of 44.3. Scores on the Montreal Cognitive Assessment ranged from 23 to 28, with a mean score of 25.8. FOG was identified in all participants during a personalized walking trajectory that was conducted at the baseline measurement of the TACKLING‐FOG trial. The mean percentage of time spent frozen was 36% (range = 0.7–96.5).

**TABLE 1:** Participant characteristics.

Participant ID	Sex	Age (y)	Years since diagnosis	Hoehn and Yahr stage	MoCA score	MDS‐UPDRS part III	NFOG‐Q	PAS	NRS	%FOG
1	Male	68	17	2	27	49	23	22	9	72.4
2	Female	70	17	3	28	46	17	14	3	25.3
3	Female	70	1	2	27	48	21	21	6	14.3
4	Male	68	9	3	26	31	22	22	8	21.3
5	Male	74	1	3	23	51	19	13	4	96.5
6	Female	45	11	2	28	43	23	19	7	55.2
7	Male	56	15	2	28	53	10	14	6	4.2
8	Female	77	12	2	24	43	22	3	4	45.1
9	Female	71	12	2	27	40	22	14	8	47.7
10	Female	78	16	2	24	47	21	22	8	17.7
11	Male	78	9	2	24	48	21	22	8	31.4
12	Male	69	3	2	23	33	19	9	6	0.7

*Note:* NRS: Numeric rating scale (0–10) indicating the extent to which anxiety or stress affected the occurrence of FOG during the past week; PAS: Parkinson Anxiety Scale; %FOG: indicates the mean percentage time frozen during a personalized walking trajectory in the home‐setting of the participants, which was part of the TACKLING‐FOG trial.

Abbreviations: MDS‐UPDRS: Movement Disorders Society—Unified Parkinson's Disease Rating Scale; MoCA: Montreal Cognitive Assessment; NFOG‐Q: New Freezing of Gait Questionnaire.

We identified five themes: Loss of confidence about one's own walking abilities (i), Regaining confidence and control while walking (ii), Gradual progression and acute surges in anxiety (iii), Directing attention toward potential threats (iv), and Avoidance and impact on daily life (v). We describe each theme below, supported by illustrative quotes from participants.

### 3.2 Theme 1: Loss of Confidence About One's Own Walking Abilities

Participants reported that FOG gradually reduced their confidence in their ability to move without difficulty, which in turn led to increased anxiety. Freezing was described as creating a sense of losing control over one's walking. Loss of confidence was also linked to the unpredictability of freezing episodes, as FOG could occur at any moment. Finally, participants reported feeling unsafe and vulnerable during walking. Each of these subthemes is described in more detail below:

#### 3.2.1 Perceived Loss of Control Over Walking

Several participants reported that FOG evoked anxiety by undermining their sense of control over movement. For example, some participants stated that they felt unsafe due to concerns about falling, which they felt unable to prevent because of FOG. Also, a participant shared that due to freezing, he wasn't sure if he could get out of a challenging situation if needed. Overall, this perceived lack of control led to distress, as it made participants feel unsafe while walking:

Yes, because, because look, at the moment when you're walking and you keep having that freezing, then you also feel that you're unsafe, that you have no control over your own body, no control over your stability and your posture. [participant 9]

#### 3.2.2 Unpredictability About Effective Movement

Some participants reported feeling anxious because FOG could happen at any moment while walking, which made them feel constantly on edge and cautious during walking:

That's. I wake up. I get up, and then I already think, ‘Oh dear, I hope this goes well.’ [participant 3]

So it's, it's figuring it out each time, a surprise how it will go. [participant 11]

One participant mentioned feeling particularly anxious at the start of walking, as he was uncertain whether he would be able to walk without problems, his anxiety decreasing once freezing did not occur:

But especially the beginning of walking, then it's really just a bit of cautiously trying and a bit anxious. And the longer you walk, the better it probably gets. [participant 7]

#### 3.2.3 Feeling Unsafe and Vulnerable While Walking

Several participants reported feeling unsafe or vulnerable because of the possibility of freezing, which was most often linked to the risk of falling during a FOG episode. However, the possibility of receiving hurtful comments related to FOG from passersby was also mentioned as contributing to the anxiety surrounding FOG.

I'm especially afraid of freezing. Afraid to stand still and have all the attention on me and … Or that people don't know me and therefore don't understand what's going on and then get angry, like ‘just keep walking.’ The confrontation. [participant 6]

One participant noted that the sense of insecurity was not just a feeling, but reflected a real risk of falling:

Then I'm just not good enough. And then I'm afraid of falling. And, that's a real risk, because with me, I always push it to the limit. [participant 2]

### 3.3 Theme 2: Regaining Confidence and Control While Walking

#### 3.3.1 Having a Backup Strategy Available

Several participants mentioned having a backup strategy in place in case FOG would occured. It was mentioned that having a strategy at one's disposal when needed alleviated anxiety as it provided a sense of control over situations in which FOG might occur. One participant actually felt this was the most effective way to reduce FOG‐related anxiety:

With my wife, I can just grab her shoulder. Then I can walk on again. So it's really like, ‘I've got something to fall back on.’ That's mainly it. I think that's the biggest stress reliever of all: I've got something to fall back on. [participant 7]

Participants mentioned various strategies they used as fallback options. First, some participants mentioned that they could use the mental strategies that they had acquired through the intervention as a fallback option, as they could use it to reduce anxiety in challenging situations, thereby making the occurrence of FOG less likely:

You're, you know … Now that you have a strategy, one that you know can work, it gives you more confidence, you know. Like, ‘Okay, I can fall back on that.’ Or actually, fall back… It's more like, I know this can help me. [participant 6]

Some noted that other types of compensation strategies also helped. As a couple of participants explained, having a compensation strategy such as consciously making a bigger step or using a cueing device enabled them to break out from a freezing episode, which provided them with a sense of control over the situation. Also, walking with someone else present and walking while carrying a walking aid were mentioned as fallback options. For example, one participant mentioned merely carrying his cane without using it made him feel less anxious during walking:

When I walk with my cane, most of the time I don't actually use it. I mean, I don't lean on it. I do have it with me, but I catch myself hardly using it, that I walk without really using the cane. […] Well, I think you feel less anxiety. You have … you always have something in your hand. Something that makes you think, ‘If anything goes wrong, I still have something to fall back on. [participant 5]

#### 3.3.2 Contextual Factors That Influence Confidence While Walking

Participants also reported contextual influences that affected their confidence while walking. Some mentioned that they felt more confident during the dopaminergic ON state, as during those moments the chance of experiencing FOG was less likely.

Well … there are moments when I feel really good. Those are the moments after you've taken your medication. And then you just walk like a lark again. And then you're also not afraid. Then, then you're completely full of confidence. And it goes well. [participant 8]

Walking in familiar and predictable environments, as well as walking in environments where the consequence of falling due to FOG was low, were also mentioned to influence FOG‐related anxiety. For example, one participant mentioned not feeling anxious when walking in spacious places because she knew the likelihood of FOG occurring was low. Several participants also started feeling less anxious when the beginning of a walk went smoothly. One participant described feeling less anxious while walking in the forest or swimming pool, indicating that he felt that the consequence of falling due to FOG was lower in those places. A few participants also mentioned feeling less anxious when physical supports, such as a robust table, were available, as these could help prevent a fall during a FOG episode.

One participant described the benefits of stability exercises and strength training for the lower extremities as a way to increase confidence while walking, thereby reducing anxiety:

“So balance exercises. And then strengthening the legs even more. Lots of leg strength training with weights. Yes, that, that is very important.” […] When asked why these exercises reduce anxiety, the participant explained: “Well, when you feel that you are stronger, and your legs shake less, you quickly think, ‘God, that's really something.’ Then you start gaining more confidence. The same goes for balance.” [participant 2]

On a final note, when asked about advice for other people with PD for how to deal with FOG, one participant explicitly emphasized the importance of finding something that boosts confidence to reduce FOG‐related anxiety:

Do your own research, stay true to yourself, and make sure, uh, that you find something that gives you confidence, restores confidence in your own body. And that confidence should help you regain some sense of control. Not like you had before, but through applying the strategy. Because that is also control. [participant 3]

### 3.4 Theme 3: Gradual Progression and Acute Surges in Anxiety

#### 3.4.1 Gradual Increase in Anxiety due to FOG‐Progression

Participants indicated that anxiety around FOG often developed gradually. Most participants were unable to recall the exact onset of FOG‐related anxiety. However, some participants noted that there was a period during which FOG was already present but anxiety had not yet emerged.

But at the beginning there was no anxiety at all. And later, the anxiety appeared. [participant 7]

FOG‐related anxiety was mentioned to develop gradually as the freezing itself progressed. For example one participant noted that a brief freeze sparked initial uncertainty, which over time snowballed into anxiety and feeling panicked during walking. This led her to ruminate on her experiences, which seemed to intensify the anxiety further:

I think it [anxiety] has built up a bit. And it started with, yeah, something small. Uh, freezing very briefly. And from that, I thought, ‘Hey, what's happening now?’ And that kept going further and further. And it made me, I think, make it much bigger myself. [participant 3]

It was also mentioned that as FOG worsened, it led to a perceived loss of control over walking, which in turn increased anxiety:

And at some point, I just kept deteriorating. And then, at one point, I noticed that my legs, that it wasn't normal anymore, they didn't respond to me anymore. (…) And yes, I started to realize that. And then I became afraid, because I thought, yes, now I've lost my only sense of security. [participant 2]

#### 3.4.2 Increase in Anxiety After Negative FOG‐Related Events

In addition, several participants reported an intensification of anxiety following negative events related to FOG. For some, anxiety gradually increased after repeated, less severe events, such as frequent falls, which led to growing concern about the possibility of injury. In contrast, a sudden surge in anxiety could occur following a single particularly distressing event. For example, one participant described a severe fall that resulted in a loss of consciousness and injury, subsequently leading to significant anxiety about falling due to FOG. Negative events were not limited to the occurrence of falls; however, receiving a hurtful comment related to a FOG episode could also trigger anxiety:

“Yes. It actually started at some point when someone was walking behind me and said, ‘Hey, keep walking, lift your feet.’ And I found that so awful. So I shouted back at that person, ‘Yeah, sorry, I can't help it. I have Parkinson's.’ And that's when the anxiety, I think, began. I thought, I don't want to experience that again.” [participant 6]

### 3.5 Theme 4: Directing Attention Toward Potential Threats

Several participants described that in situations in which they were anxious, their attention became strongly directed toward worrying thoughts and potential dangers in the environment. This focus on potential threats was described to intensify anxiety and made it more difficult to concentrate on walking itself. Some participants reported that during anxiety‐provoking situations, they felt as though too much was happening at once, with their attention competing between the perceived threat (e.g., place where they previously froze), worrisome thoughts, and the effort to focus on walking or applying their intended strategy. This attentional conflict was reported to intensify anxiety and further exacerbate FOG episodes:

Yes, it is hard to imagine. I can hardly describe it. There is so much happening in your head, and because of that, I notice that it only gets worse. [participant 6]

Several participants indicated that during stressful FOG‐related situations, their attention was drawn toward worrisome thoughts. These thoughts were often about potential negative consequences of FOG, such as the possibility of falling or ending up in a socially embarrassing situation, making it difficult to stay focused on walking:

And I also notice that I really … yes. (silence). Talk to myself very negatively, so to speak. Like, ‘Why is this happening now? Why now?’ And ‘Come on!’… (short silence) No, I don't actually say that … [participant 6]

And then it went through my mind, ‘I just hope I don't fall by the, by the, by the trash bin.’ And ‘I just hope I don't fall over those, those little thresholds and the sidewalks.’ [participant 2]

Some participants also mentioned that, when feeling anxious, their attention would focus on the source of their anxiety. For example, one participant mentioned feeling anxious in public because of being concerned about what others might think about a potential freezing episode. She mentioned being primarily focused on the people around her, instead of focusing on a strategy:

I think it's (attention) mainly towards the people and towards the anxiety. Not, not towards my breathing or anything like that, that's something I'm trying to apply now. But I think before, I was mainly focused on, uh. Uh. My attention mostly went to what was happening around me, uh, and the anxiety itself. [participant 6]

In addition, some participants reported having difficulties focusing on either walking or applying a strategy when experiencing anxiety related to FOG:

Uh. Yeah, I do think there's a reason that when I'm stressed, I tend to freeze more easily. It's, uh, the problem. Let me call it a problem. I'm so focused on it that I, uh… that… my attention is no longer on other things… [participant 4]

But I have to be very consciously calm. ‘Calm steps,’ ‘calm walking,’ ‘calm thinking.’ I have to, I quickly get stressed about, uh, this needs to happen, that needs to happen. And then I'm not focused. And then I freeze again [participant 2]

### 3.6 Theme 5: Avoidance and Impact on Daily Life

Several participants reported that they started to avoid activities due to anxiety surrounding FOG. Some avoided activities by replacing walking with alternatives. For example, one participant explained that he started contacting colleagues by phone rather than walking over to them. Another participant shared that she moved herself around in the classroom using a desk chair instead of walking. Participants' avoidance behavior influenced multiple aspects of their daily activities, encompassing work‐related activities, daily activities such as grocery shopping, and doing volunteer work:

I didn't go to last year's annual trip to the flea market because I was afraid I would experience freezing. [participant 6]

And, and another point is, I still sometimes have that. That I then want to go do grocery shopping, or have to do so, but then I don't get to it for a year, no, weeks, no, sorry, days. Then I'm still not good enough. And then I'm still afraid of falling. [participant 2]

Participants described that avoiding walking had a number of negative consequences. First, some participants noted that avoidance led to feelings of loneliness, since it led them to leave the house less often.

Well, so, uh, if you are influenced by, being outside, then you prefer to stay in your own cocoon. Because there you feel safe. And then there, you have peace of mind. But the consequence of that is that you end up lonely. [participant 10]

Also, physical inactivity was mentioned as a negative consequence that was due to avoidance behavior.

Yes, that you did things more slowly. That you stayed seated longer and did nothing, that you just say, ‘I better not do that because maybe I'll fall, or maybe I'll come across something that's frightening.’ So then you avoided those situations. [participant 5]

Last, avoidance led some participants to stop doing activities that they found meaningful or pleasant, such as doing volunteer work, going to concerts, or spending time with friends.

And I also avoid. Nowadays I don't go to concerts anymore. At least, certainly not now … I used to, I used to really enjoy going to concerts. The last concert was really a disaster. And then I said to myself, ‘I'm not going, not going to a concert anymore. [participant 7]

Interestingly, some participants mentioned that the strategies they acquired through the intervention led them to engage in situations they previously avoided, as one participant noted:

“I've noticed that now I go to more situations, situations. That I go to more situation where people are. And I feel less anxious, so to speak. I do a lot more. Just ask my boyfriend, he's been at home for a while now, and he says, ‘You're out quite often.’ And before… since March he's been at home for a while, so he noticed and said, ‘Yes, now you go out more often.’ Without thinking, ‘Okay, you go, because I don't want to experience that situation,’ or ‘I want to prevent freezing.’ I also feel that I'm a bit less focused on others.” [participant 6]

## 4 Discussion

This study applied a qualitative approach to enhance our understanding of how people with PD experience and reflect on the interaction between anxiety and FOG following participation in a behavioral intervention. In doing so, we aimed to gain insight into how individuals with PD describe and reflect on the development of anxiety related to FOG and its impact on their daily lives.

**FIGURE 1:**
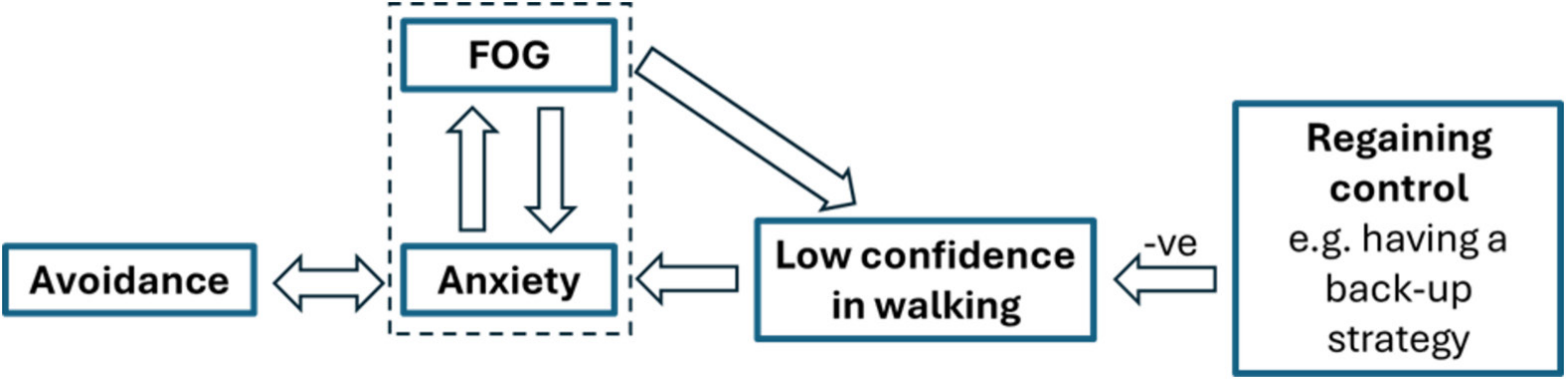
Proposed framework depicting how anxiety, FOG, and walking confidence are interrelated.

Our findings indicate that participants described anxiety surrounding FOG as emerging alongside a loss of confidence in their ability to walk effectively (Figure 1), which was driven by a perceived loss of control over their walking, unpredictability of FOG episodes, and feeling unsafe and vulnerable during walking. This loss of confidence during walking may cause anticipatory anxiety, as individuals must remain vigilant for potentially harmful FOG episodes. Having low confidence during walking might also undermine the belief of people with PD in their ability to effectively respond to FOG episodes, which closely relates to research showing that self‐efficacy—that is, one's perceived ability to succeed in a particular situation—strongly contributes to perceived walking difficulties in people with PD (Kader et al. 2017). Related to the loss of perceived control during walking, previous work has described the relationship between perceived control and anxiety about falling (Ellmers, Wilson, et al. 2023; Ellmers et al. 2022). According to the perceived control model of falling, encountering a potential threat prompts an appraisal of both the anticipated harm and the likelihood of that harm occurring, which in turn leads to conscious behavioral adaptations aimed at reducing fall risk (Ellmers, Wilson, et al. 2023). When perceived control is low, however, individuals may experience heightened anxiety, characterized by worrisome thoughts that further increase the risk of falling. Applied to anxiety surrounding FOG, this framework highlights the potential value of interventions that enhance perceived control, thereby reducing anxiety, interrupting maladaptive threat responses, and supporting more adaptive strategies for managing FOG.

Participants described several ways of regaining confidence while walking, which in turn helped reduce anxiety related to FOG. In this regard, several participants noted that having a backup strategy available in case FOG occurs effectively reduced anxiety around FOG. The notion that having a backup plan available can help reduce FOG‐related anxiety has been previously described (Nonnekes et al. 2019). For instance, one patient experienced improvements in his FOG while wearing laser shoes, even though he did not actually look at the projected laser beams while walking (Nonnekes et al. 2019; Ferraye et al. 2016). He reported that simply having the shoes available made him feel more comfortable. Another example involved a man whose FOG worsened in poorly lit environments (Goossens et al. 2025). Notably, he carried a cane that he used only in the dark, indicating that he primarily relied on it as a fallback strategy, thereby lowering anxiety. Contextual influences that were reported to affect anxiety related to FOG, such as being in the dopaminergic ON state or walking in familiar environments, may alleviate anxiety by reducing unpredictability during walking and lowering the perceived likelihood of harm occurring. In addition to these context‐specific effects, previous studies have already reported that dopaminergic medication can have anxiolytic effects in patients with PD (Maricle et al. 1995). It is important to emphasize that some degree of perceived lack of control can be adaptive, as it enables individuals to anticipate potential risks and adopt careful movement strategies to prevent FOG‐related falls (Landers and Nilsson 2023).

Participants described FOG‐related anxiety as developing progressively over time, driven by both increasing FOG severity and negative FOG‐related experiences. While previous research has shown that anxiety can predict subsequent FOG onset in PD (Ehgoetz Martens et al. 2018), our results further suggest that FOG itself may contribute to the development of anxiety. As FOG progresses, individuals with PD appear to become increasingly insecure while walking and more frequently encounter adverse consequences of FOG episodes. However, susceptibility to developing FOG‐related anxiety likely varies between individuals and may be influenced by underlying psychological vulnerabilities, such as elevated trait neuroticism (Widiger and Crego 2019). Moreover, while anxiety appears to play a central role in FOG for the participants in this study, it is important to acknowledge that in other individuals with PD, anxiety might play a more limited role in the occurrence or experience of FOG (Martens et al. 2018). Importantly, negative FOG‐related experiences were associated with an acute increase in FOG‐related anxiety. This may reflect associative learning processes consistent with fear conditioning, in which FOG becomes paired with the threat of aversive outcomes such as falling (Lissek et al. 2005). Consequently, individuals may develop anticipatory anxiety about FOG. This mechanism has previously been proposed in relation to fear of falling (Peeters et al. 2020). Beyond these learning mechanisms, the content of FOG‐related anxiety may also be more complex than fear of falling alone. Anxiety related to fear of falling and FOG overlap, as episodes of FOG may evoke concerns about falling and potential injury. However, FOG‐related anxiety appears broader in scope, encompassing not only fear of falling but also anxiety related to the unpredictability of FOG and the inability to initiate or continue walking. Taken together, these findings underscore the importance of early intervention to prevent or mitigate the development of FOG‐related anxiety as FOG progresses. Notably, when asked to provide advice to others with PD, one participant explicitly highlighted the value of early education and the acquisition of strategies to reduce uncertainty surrounding FOG.

Our results also indicate that when experiencing anxiety, attentional resources tend to be directed toward information related to potential threats, making it more difficult to focus on a selected compensation strategy. Previous work has similarly proposed that anxiety leads to the preferential processing of threat‐related information at the expense of goal‐directed motor control (Rosenblum et al. 2025). These anxiety‐related attentional changes may be counteracted by deliberately reorienting attention. Whether deliberately reallocating attention away from gait‐related threatening stimuli and toward a behavioral strategy can counteract these changes is currently being investigated in the ongoing TACKLING‐FOG trial (Vissers et al. 2026).

Lastly, anxiety surrounding FOG was reported to lead individuals to avoid situations that might trigger freezing, which had several negative downstream effects, including reduced participation in meaningful and enjoyable activities, physical inactivity, and social isolation. Previous work has suggested that FOG is one of the strongest predictors of fear of falling‐related avoidance behavior (Bloem et al. 2004). Interestingly, the acquisition of compensation strategies led some individuals to increase engagement in daily activities, potentially reflecting a regained confidence in walking. This increased confidence may, in turn, have reduced anxiety and ultimately contributed to a decrease in avoidance behavior. While compensation strategies are typically discussed in terms of their benefits to gait performance, our findings suggest that their impact may extend to confidence and activity engagement. Future work should further explore this relationship. Finally, it is important to note that avoidance can serve both adaptive and maladaptive functions. (Landers and Nilsson 2023; Ellmers, Freiberger, et al. 2023) On one hand, avoidance can help individuals to keep away from risky situations, thereby increasing safety. Conversely, avoidance can become maladaptive when it is disproportionate, severely limits functional behavior, or reduces long‐term well‐being and safety. (Landers and Nilsson 2023; Hadjistavropoulos et al. 2011) This underscores the importance of assessing and targeting maladaptive avoidance that is related to FOG, to maintain functional independence and overall well‐being in people with PD.

This study was not without shortcomings. First, interviews were conducted during the final session of the intervention. While this timing provided a contextual backdrop for participants to reflect on their experiences, potentially facilitating more detailed descriptions, it may also have influenced participants' responses. Specifically, the timing may have introduced recall bias, as participants' accounts could have been shaped by their increased reflection and reinterpretation of experiences during the intervention period. Future research could build on this work by including participants prior to intervention, allowing for comparison of perspectives at different stages of awareness and reducing the influence of structured reflection. In addition, the timing of the interviews would have been appropriate for eliciting feedback on the intervention itself, which could have provided additional insights for optimizing the clinical protocol. Another limitation of the study was that we only included participants who experienced daily episodes of FOG, which limited the exploration of anxiety in individuals in the early stages of freezing. Additionally, the prioritization of participants who were able to articulate their experiences in detail may have introduced a degree of bias toward more verbally expressive individuals, potentially affecting the breadth of perspectives captured. A last limitation is that member checking was not performed, meaning participants did not have the opportunity to review and confirm the findings.

We conclude by highlighting some clinical implications of this study. First, our findings emphasize the importance for people with PD of identifying strategies or factors that enhance confidence while walking, as this may help maintain mobility and reduce the risk of FOG‐related falls. Second, early recognition and intervention for anxiety related to FOG, and FOG itself, are crucial to prevent escalation of anxiety and to reduce the risk of a vicious cycle between anxiety and FOG. Third, providing patients with education on how anxiety can influence attention in challenging walking situations may help them manage these moments more effectively. Lastly, addressing avoidance around FOG is important to preserve functional independence and quality of life as much as possible.

## Author Contributions

**Gijs Vissers:** conceptualization, writing – original draft, methodology, software, formal analysis, visualization, investigation. **William R. Young:** conceptualization, writing – review and editing, supervision, visualization. **Jorik Nonnekes:** conceptualization, writing – review and editing, supervision.

## Funding

This study was conducted as part of the TACKLING‐FOG trial, funded by the Jacques and Gloria Gossweiler Foundation.

## Ethics Statement

Ethical approval was obtained from the Medical Ethics Committee Oost‐Nederland, the Netherlands, as part of the TACKLING‐FOG trial, and the research was carried out in accordance with the Declaration of Helsinki.

## Conflicts of Interest

The authors declare no conflicts interest.

## Supporting information


**Data S1:** Supporting Information.

## Data Availability

The data of this study will be available from the corresponding author upon reasonable request once the findings of the study are published.
