# Supplementary material for: Experiences of Anxiety and Its Relationship to Freezing of Gait in Parkinson's Disease: A Qualitative Study
Source: Eur J Neurosci. 2026 Jun 17;63(12):e70581. doi: 10.1111/ejn.70581 (PMC13276019; doi:10.1111/ejn.70581)
Supplement: Supplementary file 1 — Data S1: Supporting Information. [file EJN-63-0-s001.docx]

**Supplementary material**

**Interview Guide**

Part 1: General questions about Anxiety around Freezing
*I would like to ask you some questions about how you experience anxiety related to freezing of gait. I would like to ask you to describe your personal experiences.*

1. In what situations do you experience anxiety related to freezing of gait? (explore each situation in detail)
2. What do you think causes you to feel anxiety in this situation? (explore per situation)
3. In situation X, can you tell me what is going through your mind at that moment? (go through each situation)
4. Are there certain situations in which you do not feel anxiety, even if freezing could occur? Why?
5. Can you describe how anxiety about freezing affects your daily life? Have you started doing things differently than before?
6. What happens to your attention when you feel anxious while walking?

Part 2: Effect of Strategy on Anxiety and Freezing

1. Does having a strategy affect your anxiety while walking? Can you tell me more about that?
2. Some people say that having a strategy (or aid such as a walking stick or walker) sometimes helps them walk better, even at times when they are not actively using the strategy. Do you recognize this?

If yes, do you have an idea why this works?

1. Does anxiety affect your ability to focus on walking or use a strategy? How?

Part 3: Course of Anxiety around Freezing
*I am also curious about how your experience with anxiety around freezing developed and changed over time.*

1. Can you tell me something about how your anxiety around freezing has developed over time or how it originated?
2. Has there been a situation that had a big impact on your anxiety about freezing?
3. Can you remember when you first experienced anxiety related to freezing?
4. Has your anxiety about freezing changed over time, for example, has it become worse or better?

Part 4: Final questions

1. Do you have tips for other people with Parkinson’s to deal with freezing of gait?
2. Is there anything we haven’t talked about yet that you would like to add?
